# Cervical intraepithelial neoplasia disease progression is associated with increased vaginal microbiome diversity

**DOI:** 10.1038/srep16865

**Published:** 2015-11-17

**Authors:** A. Mitra, D. A. MacIntyre, Y. S. Lee, A. Smith, J. R. Marchesi, B. Lehne, R. Bhatia, D. Lyons, E. Paraskevaidis, J. V. Li, E. Holmes, J. K. Nicholson, P. R. Bennett, M. Kyrgiou

**Affiliations:** 1 Institute of Reproductive and Developmental Biology, Department of Surgery & Cancer, Imperial College, London, UK; 2 Department of Obstetrics and Gynaecology, Queen Charlotte’s & Chelsea – Hammersmith Hospital, Imperial Healthcare NHS Trust, London, UK; 3 School of Biosciences, Cardiff University, CF10 3AX, UK; 4 Section of Biomolecular Medicine, Division of Computational Systems Medicine, Department of Surgery and Cancer, Imperial College London, UK; 5 Department of Epidemiology & Biostatistics, Medicine, Imperial College London, UK; 6 HPV Research Group, Division of Pathology, University of Edinburgh, UK; 7 Department of Obstetrics and Gynaecology, St Mary’s Hospital, Imperial Healthcare NHS Trust, London, UK; 8 Department of Obstetrics and Gynaecology, University Hospital of Ioannina, Ioannina, Greece

## Abstract

Persistent infection with oncogenic Human Papillomavirus (HPV) is necessary for cervical carcinogenesis. Although evidence suggests that the vaginal microbiome plays a functional role in the persistence or regression of HPV infections, this has yet to be described in women with cervical intra-epithelial neoplasia (CIN). We hypothesised that increasing microbiome diversity is associated with increasing CIN severity. llumina MiSeq sequencing of 16S rRNA gene amplicons was used to characterise the vaginal microbiota of women with low-grade squamous intra-epithelial lesions (LSIL; n = 52), high-grade (HSIL; n = 92), invasive cervical cancer (ICC; n = 5) and healthy controls (n = 20). Hierarchical clustering analysis revealed an increased prevalence of microbiomes characterised by high-diversity and low levels of Lactobacillus spp. (community state type-CST IV) with increasing disease severity, irrespective of HPV status (Normal = 2/20,10%; LSIL = 11/52,21%; HSIL = 25/92,27%; ICC = 2/5,40%). Increasing disease severity was associated with decreasing relative abundance of Lactobacillus spp. The vaginal microbiome in HSIL was characterised by higher levels of Sneathia sanguinegens (P < 0.01), Anaerococcus tetradius (P < 0.05) and Peptostreptococcus anaerobius (P < 0.05) and lower levels of Lactobacillus jensenii (P < 0.01) compared to LSIL. Our results suggest advancing CIN disease severity is associated with increasing vaginal microbiota diversity and may be involved in regulating viral persistence and disease progression.

Persistent infection with a high-risk oncogenic Human Papillomavirus (HPV) subtypes, most commonly 16 and 18, is a necessary, although not sufficient, condition for development of invasive cervical cancer (ICC) and its precancerous precursor; cervical intra-epithelial neoplasia (CIN)^1^. Although HPV infection is very common in sexually-active women^2^, the majority of infections are transient^3^. Only a small proportion of women infected with the virus goes on to develop clinically significant pre-invasive lesions and, if not treated, invasive malignant disease. Mechanisms of persistence of HPV infection are not well understood.

The vaginal microenvironment plays an important role in reproductive health. Commensal vaginal *Lactobacillus* spp. are thought to defend against pathogens and sexually transmitted infections^4^ through maintenance of a hostile pH^5^, production of species-specific metabolites, bacteriocins and through adherence to mucous and disruption of biofilms^6^,^7^,^8^,^9^. Next generation sequencing (NGS) based studies have facilitated detailed characterisation of the “healthy” vaginal microbiome and shown that 5 major community-state types (CSTs) exist; CST I, II, III and V are dominated by *Lactobacillus crispatus, L. gasseri, L. iners* and *L. jensenii* respectively, whereas CST IV has characteristically low numbers of *Lactobacillus* spp. and increased diversity of anaerobic bacteria^10^. Longitudinal studies of the vaginal microbiome using NGS indicates that bacterial community structure is dynamic and hormonally influenced with a propensity to become less stable during menstruation^11^ and conversely more stable and less diverse during normal pregnancy^12^,^13^. The stability and composition of the vaginal microbiome may play an important role in determining host innate immune response and susceptibility to infection. Bacterial vaginosis (BV), a condition characterised by *Lactobacillus* spp. depletion, overgrowth of anaerobic species, and higher vaginal pH has been associated with increased transmission rates of sexually-transmitted infections^14^ and human immunodeficiency virus (HIV)^15^. Conversely, it has recently been reported that viral infection of the cervix during murine pregnancy increases susceptibility of ascending vaginal bacterial infection through sensitisation and priming of the host innate immune system^16^.

Relatively little is known about the mechanisms associated with clearance or persistence of HPV infection. Along with higher rates of HPV infection, BV has been associated with delayed clearance of the virus and with CIN, suggesting that a diverse, *Lactobacillus*-depleted microbiome may play a mechanistic role^17^,^18^,^19^,^20^. A recent study of 68 HPV-discordant monozygotic female Korean twins using NGS showed that HPV-positive twins had lower levels of *Lactobacillus* spp. and increased counts of *Fusobacteria* and *Sneathia* spp. compared to their HPV-negative twins^21^. Consistent with these findings, analysis of vaginal swabs collected longitudinally for 16 weeks from 32 sexually active women found that a *Lactobacillus* spp.-depleted, *Atopobium* spp. enriched (CST IV) community structure is associated with slowest regression of HPV whereas a *Lactobacillus gasseri*-dominated microbiome (CST II) is associated with the most rapid regression rates for HPV^22^.

While there is evidence of an association between vaginal microbiome structure and HPV infection, the potential relationship between the vaginal microbiota and CIN disease progression has yet to be investigated. In this study, we characterised the vaginal microbiome structure and diversity in women with pre-invasive cervical intraepithelial neoplasia, invasive cervical cancer, and in healthy controls to assess how CSTs may correlate with disease presence and severity. We hypothesised that increasing microbiome diversity is associated with increasing CIN severity.

## Results

We enrolled 169 women into the study who were classified into 4 groups; normal (n = 20), low-grade squamous intraepithelial lesion (LSIL) (n = 52), high-grade squamous intraepithelial lesion (HSIL) (n = 92) and ICC (n = 5). Table 1 shows the characteristics of each group. No difference in the mean age of the population was determined (mean 31, SD 5.08, range 23–45, *P* = 0.071). No category showed systematic bias with respect to the four disease-groups after adjustment for multiple hypothesis testing. There was equal distribution in the samples collected at the follicular or luteal phase of the cycle and in the rate of women that had intercourse within 48 hours from sample collection. Histology was available in all HSIL and cancer cases (100%), and for 69% (36/52) and 5% (1/20) of LSIL and normal cases, respectively.

### The structure of the vaginal microbiome correlated to the disease severity

In total 8 409 192 reads were obtained from 169 samples with an average number of reads per sample of 49 759 and the mean and median read lengths of 513 and 520 bp respectively. To avoid sequencing bias, operational taxonomic units (OTUs) were randomly sub-sampled to the lowest read count of 183, which retained 97% of OTU counts (data not shown) and still provided coverage of >96% for all samples. Following removal of singletons and rare OTUs, a total of 49 taxa were identified in the vaginal microbiome of the study cohort.

**Figure 1:**
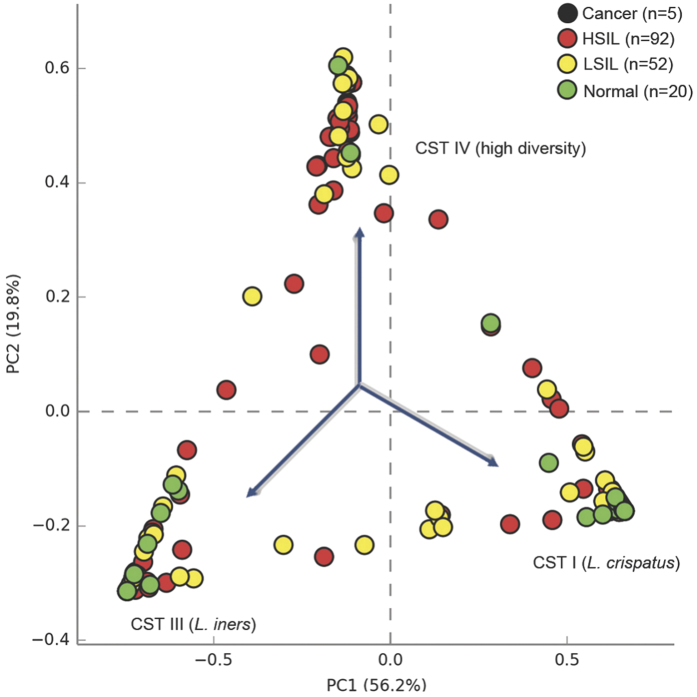
Bacterial species diversity in study cohort and controls. Principle component analysis (PCA) of vaginal bacterial species data identified 3 major clusters corresponding to samples dominated by three community state types (CSTs): *L. iners* (CST III), *L. crispatus* (CST I) and high diversity samples (CST IV). KEY - Cancer: Black; High-grade squamous intra-epithelial lesions (HSIL): Red; Low-grade squamous intra-epithelial lesions (LSIL): Yellow; Normal: Green.

Initial assessment of vaginal community structure was performed using principal component analysis (PCA) of species sequence data in the context of disease grade (normal, LSIL, HSIL and ICC) (Fig. 1). Three major clusters were identified which represented samples dominated by either *L. crispatus, L. iners* or samples depleted of *Lactobacillus* spp. with higher diversity. The results of the analysis at class level are presented in Supplementary Figure 1.

**Figure 2:**
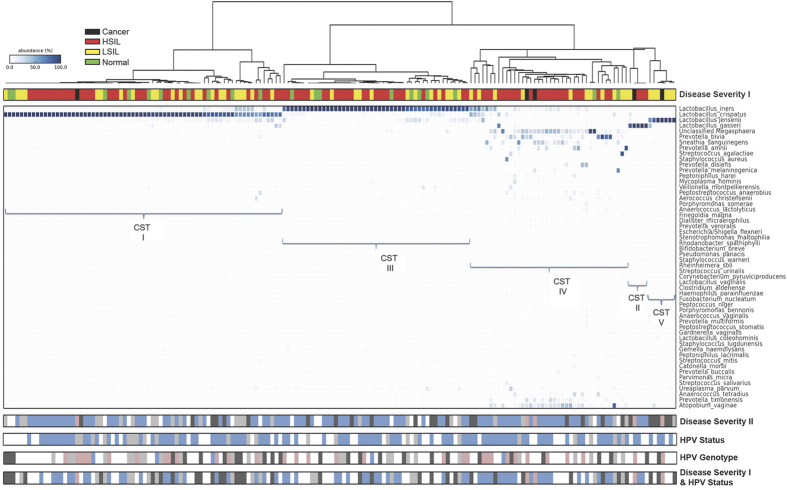
Vaginal microbiome composition according to disease and HPV status. Hierarchical clustering analysis (HCA) using centroid clustering showed the distribution of CSTs differs in healthy, normal control women (green), compared to those with disease (Disease Severity I). Frequency of CST IV was 2-fold greater in women with LSIL (yellow), 3-fold greater in HSIL (red) and 4-fold greater in women with ICC (black), compared to controls, with a reciprocal decrease in frequency of CST I with increasing disease severity. When HPV/ASCUS (light grey) and LSIL (dark grey) were examined as two separate groups, the stepwise increase in CST IV prevalence was maintained (Disease Severity II). HPV positivity (blue) was associated with increased rates of CST IV, compared to HPV negative women (light grey) who were most likely to have CST I (HPV status). HPV-16 (pink) was most frequently associated with CST IV (HPV genotype). Rates of CST IV were similar in women with normal or LSIL regardless of HPV status (negative = light grey, positive = dark grey), and were substantially higher in women with HSIL and positive for HPV (blue) (Disease severity I & HPV status). DISEASE SEVERITY I - Normal: Green; Low-grade squamous intra-epithelial lesions (LSIL): Yellow; High-grade squamous intra-epithelial lesions (HSIL): Red; Cancer: Black. DISEASE SEVERITY II - Normal: white; ASCUS: light grey; LSIL: dark grey; HSIL: blue; Cancer: pink. HPV STATUS - HPV negative: light grey; HPV positive: blue; Unknown: white. HPV GENOTYPE - HPV negative: light grey; HPV16: pink; HPV18: blue; Other HR-HPV: dark grey; Unknown: white. DISEASE SEVERITY I & HPV STATUS - LSIL/Normal HPV negative: light grey; LSIL/Normal HPV positive: dark grey; HSIL/HPV positive: blue; Cancer: pink; unknown/other: white. KEY - ASCUS: atypical squamous cells of undetermined significance; CST: community state type; HPV: Human Papillomavirus; HR-HPV: high-risk HPV; HSIL: High-grade squamous intra-epithelial lesion; ICC: invasive cervical cancer; LSIL: Low-grade squamous intra-epithelial lesion.

Hierarchical clustering analysis (HCA) of the sequence data using nearest neighbour linkage at species level (Fig. 2) identified 5 major clusters that exhibited bacterial community structure consistent with previously described vaginal microbiome community state types (CSTs); CST I: *L. crispatus*-dominated, CST II: *L. gasseri*-dominated, CST III: *L. iners*-dominated, CST IV: *Lactobacillus*-depleted and CST V: *L. jensenii*-dominated^10^ (Fig. 2). The results of the analysis at class level are presented in Supplementary Figure 2 & Supplementary Table 1.

The rates and frequency of the different CSTs (I, II, III, IV & V) were compared between CIN disease severities and healthy controls (Table 2; Supplementary Table 3; Fig. 2). CST I was the most frequent CST in our study cohort (70/169, 41% of all patients), followed by CST III (47/169, 28%), CST IV (40/169, 24%), CST V (7/169, 4%) and CST II (5/169, 3%). Higher rates of CST IV (*Lactobacillus*-depleted, high diversity) were associated with increasing disease severity with CST IV observed twice as frequently in women with LSIL, three times as frequently in women with HSIL and four times as frequently in women with ICC when compared to disease free controls (normal = 2/20, 10%; LSIL = 11/52, 21%; HSIL = 25/92, 27%; cancer = 2/5, 40%). Conversely, frequency of CST I (*Lactobacillus crispatus*-dominant) was lower with increasing disease severity (normal = 10/20, 50%; LSIL = 22/52, 42%; HSIL = 37/92, 40%; cancer = 1/5, 20%). The number of ICC cases was small for any valid conclusion. Although the results of the analysis did not attain significance given the modest sample size, there appears to be a correlation of CST IV and increasing disease severity.

A similar distribution of CST IV was observed when women with HPV/Atypical squamous cells of undetermined significance (ASCUS) and LSIL changes were analysed as two separate groups (normal = 2/20, 10%; ASCUS = 5/26, 19%; LSIL = 6/26, 23%; HSIL = 25/92, 27%; cancer = 2/5, 40%) (Table 2; Fig. 2; Supplementary Table 3). These analyses are suggestive of association between CST IV and increasing disease severity, and are consistent with the expected direction of effect, but do not attain significance due to modest sample-size.

**Figure 3:**
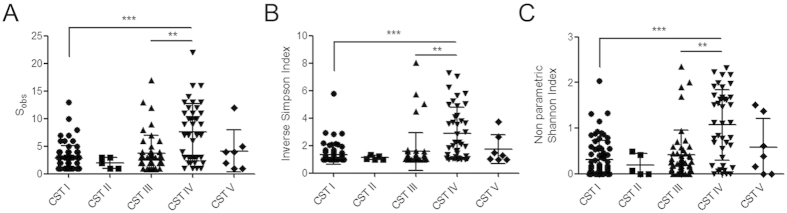
Analysis of richness (A) and diversity indices (B&C) with attributed CSTs for the patient cohort. A significantly higher number of species observed in samples classified as CST IV (**A**) compared to CST I (*P* < 0.001) and CST III (*P* < 0.01). Diversity was also significantly higher in CST IV classified samples as assessed by the Inverse Simpson (**B**) and non-parametric Shannon (**C**) indices compared to CST I (*P* < 0.001) and CST III (*P* < 0.01). Kruskall-Wallis test (Dunn’s *post hoc*). KEY - CST: Community state type; S_obs_: Species observed; ** = P < 0.01; *** = P < 0.001.

**Figure 4:**
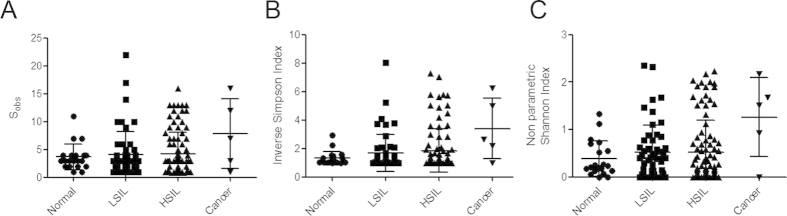
Vaginal microbiome richness and diversity indices associated with disease status (normal, LSIL, HSIL and cervical cancer patients). The number of species observed increased with disease severity with lowest richness observed in healthy controls and highest in HSIL and ICC (**A**). Diversity, as assessed by Inverse Simpson (**B**) and non-parametric Shannon (**C**) indices followed the same pattern. KEY - HSIL: High-grade squamous intra-epithelial lesion; ICC: invasive cervical cancer; LSIL: Low-grade squamous intra-epithelial lesion; S_obs_: Species observed; ** = P < 0.01; *** = P < 0.001.

Both species richness (Fig. 3A) and alpha-diversity (Fig. 3B,C) indices were higher in CST IV, compared to the other CSTs particularly CST I (P < 0.001) and CST III (*P* < 0.01) (Fig. 3). Consistent with increased rates of CST IV in high grade disease, vaginal microbiota richness and diversity were also found to be higher in women with high-grade disease, compared to low-grade disease, and lowest in normal women but this was not statistically significant (Fig. 4) (Supplementary Table 4).

### The structure of the vaginal microbiome correlated to the HPV status and genotype

HPV status was available for 117 women in our cohort. CST IV was most frequently observed in high-risk HPV (HR-HPV) positive as compared to HR-HPV negative women (26/93, 28% versus 5/24, 21%). HPV negative women were most likely to have CST I (13/24, 54%) as opposed to HPV positive women (38/93, 41%). The rates for the other CSTs were comparable between HR-HPV positive and negative subjects (Table 2; Fig. 2).

Of 93 women who were HPV positive, genotyping was available for 62 subjects. The rate of CST IV was higher for women infected with HPV16 (9/31, 29%) when compared to HPV18 (1/5, 20%) or women with other high-risk oncogenic types (5/26, 19%) although did not reach significance, likely due to sample size (Table 2; Fig. 2).

The rate of CST IV was no different for Normal/LSIL HPV negative versus HPV positive individuals (3/20, 15% versus 7/34, 21%), but substantially higher for HSIL or worse (HSIL = 19/58, 33%; ICC = 2/5, 40%), suggesting that the presence of a high diversity Lactobacillus-depleted microbiome may be more strongly correlated to the presence of clinically significant pre- or invasive disease rather than the presence of the virus itself (Table 2; Supplementary Table 3; Fig. 2). Again, the results did not reach statistical significance.

### Identification of vaginal microbiota composition markers of CIN disease severity

**Figure 5:**
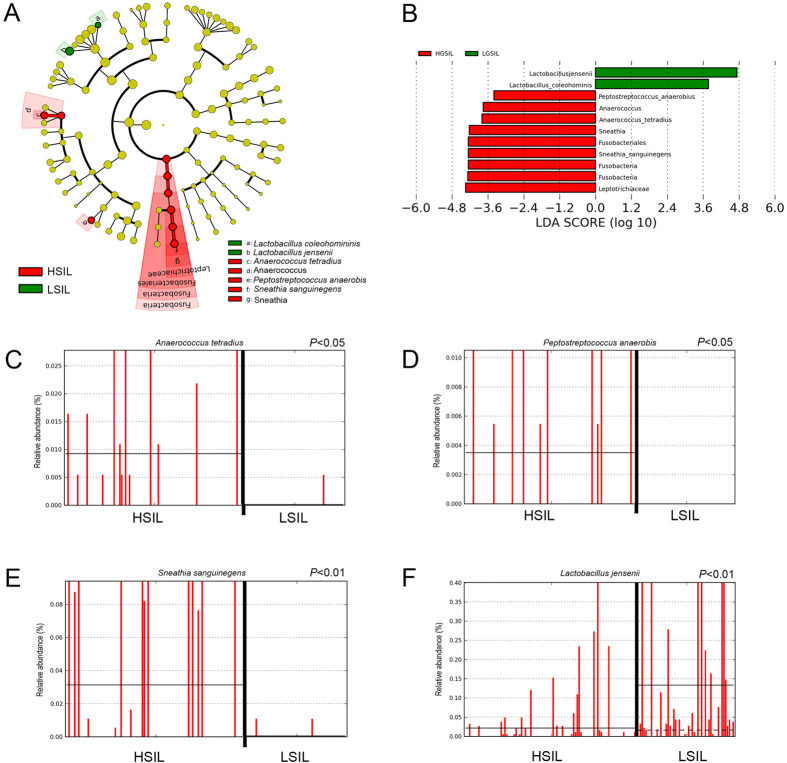
Identification of vaginal microbiota biomarkers of LSIL vs. HSIL by LEfSe analysis. (**A**) Cladogram representing taxa with different abundance according to disease severity. Size of circle is proportionate to abundance of taxon. (**B**) Histogram of the LDA scores computed for features differentially abundant between LSIL and HSIL disease states. Relative abundance counts of *Anaerococcus tetradius* (**C**), *Peptostreptococcus anaerobis* (**D**) and *Sneathia sanguinegens* (**E**), which were found to be significantly over-represented in HSIL whereas Lactobacillus jensenii (**F**) was enriched in LSIL samples (Welch’s t-test). KEY - HSIL: High-grade squamous intra-epithelial lesion; LDA score: Linear discriminant analysis score; LSIL: Low-grade squamous intra-epithelial lesion.

Linear discriminant analysis (LDA) effect size (LEfSe) modeling was used to identify differences in microbiota composition that may be related to increasing disease severity (Fig. 5). Due to sample size restrictions, we limited our comparison to LSIL versus HSIL patients. In the LSIL group, significant over-representation of *Lactobacillus jensenii* (*P* < 0.01) (Fig. 5A,B,F) and *Lactobacillus coleohominis* (*P* < 0.05) (Fig. 5B) were observed. In contrast, HSIL samples were found to have significantly higher levels of *Peptostreptococcus anaerobius* (*P* < 0.05), and *Anaerococcus tetradius* (*P* < 0.05) (Fig. 5A–D). HSIL samples were also found to have significant overrepresentation of *Fusobacteria*- primarily *Sneathia sanguinegens* (*P* < 0.01) (Fig. 5A,B,E; Supplementary Figure 3).

## Discussion

We detected a two-fold increase in the rate of a CST IV vaginal microbiome in those women with LSIL, a three-fold increase in women with HSIL and a four-fold increase in women with invasive cancer compared to controls. Increasing disease severity was also associated with decreasing relative abundance of *Lactobacillus* spp. A recent longitudinal study by Brotman *et al*. reported that women with a high diversity, *Lactobacillus* spp. depleted (CST IV) vaginal microbiome were most likely to become HPV-positive, and to have persistent HPV infection^22^. Our findings suggest that vaginal microbial diversity is associated not only with HPV infection, but also with advancing CIN severity, but does not attain significance due to modest sample-size.

It is currently unclear if a CST IV microbiome is a causal factor in progression of CIN or a consequence of it. BV, a condition diagnosed using traditional culture techniques, in part by *Lactobacillus* spp. depletion and increased diversity of potentially pathogenic gram negative bacteria, is associated with significantly higher rates of HPV infection and CIN^19^,^20^. We used NGS techniques to further examine this association and identified *Sneathia sanguinegens* as a biomarker of HSIL, which has previously been shown to associate with HPV infection^21^. Two other BV-associated bacteria; *Peptostreptococcus anaerobius* and *Anaerococcus tetradius* were also found to be markers of HSIL in our cohort. This further suggests that specific anaerobic species may play a role in disease progression, rather than simply signifying presence of HPV infection.

Bacteria are increasingly appreciated as a key player in the initiation and progression of other malignancies including colorectal cancer^23^,^24^,^25^ where *Fusobacteria* has been identified as a potential pro-carcinogenic bacterial class^25^,^26^. Our findings show that this class, and specifically *Sneathia sanguinegens*, is discriminatory of HSIL suggesting similar mechanisms, likely involving activation of inflammatory pathways, may be involved in the cervix. Approximately one third of premalignant lesions go on to develop invasive cervical disease, if untreated. It is possible that the women in our cohort with CST IV microbiomes are those at highest risk of progression to clinically significant invasive lesions, yet our findings only demonstrate association, not causality, between cervical pre-cancer, persistent HPV infection and the structure of the vaginal microbiota. Whilst women with BV display higher rates of HPV infection^14^,^19^ the virus has been shown to induce a pro-inflammatory environment to facilitate integration of viral DNA^27^,^28^,^29^,^30^,^31^. Thus HPV infection itself may adversely impact on the host’s immune defences and mucosal metabolism leading to aberration of vaginal microbiota, thus promoting viral persistence and disease progression.

*Lactobacillus* spp. are classically regarded as ‘protective’, yet our study supports other previous reports which suggest the clinical picture may be dictated by the specific species present, and that the genus as a whole may not be regarded as protective in its entirety. Brotman and colleagues reported that an *L. iners* dominated vaginal microbiome (CST III) was associated with HPV-infection whereas vaginal microbiomes dominated by *L. gasseri* (CST II) exhibited the most rapid clearance of HPV infection^22^. While we did not observe over-representation of CST II or III in our CIN cohorts, the reduced prevalence of *L. iners* (CST III) concurrent with increased rates of CST IV in CIN, compared to controls may represents a shift from *L. iners* (CST III) towards a CST IV type microbiome with the acquisition of CIN. Unlike the majority of *Lactobacillus* spp., *L. iners* does not produce H_2_O_2_, which has been shown to have antibacterial and antiviral properties^32^,^33^,^34^. Consistent with the possibility that H_2_O_2_-producing *Lactobacillus* spp. are protective against CIN progression, we detected higher prevalence of *L. jensenii* and *L. coleohominis*, both H_2_O_2_-producing lactobacilli, in women with LSIL compared to HSIL, suggests this species may be particularly protective in preventing progression of the dysplastic and ultimately carcinogenic process. Furthermore *Lactobacilli* spp., have been shown to be cytotoxic when co-cultured with cervical cancer cells *in vitro*, but not normal cells, independent of lactic acid concentrations, highlighting interactions amongst cervical cells, the microbiota and the mucosal metabolic milieu^35^.

Environmental and hormonal factors are also known to modulate the vaginal microbiome. Smoking has been previously correlated to HPV persistence and CIN as well as *Lactobacillus* spp. depletion and dysbiosis^36^. Although women in our study with high-grade disease were more likely to be smokers, the differences did not reach statistical significance and no correlation between vaginal microbial composition and smoking status was identified. A sub-analysis of smokers showed that the prevalence of CST IV increased with disease severity indicating that a high-diversity microbiome is correlated to disease status rather than smoking as a potential confounder.

Future therapeutic strategies permitting the modulation of the vaginal microbiome with oral or topical regimes to a *Lactobacillus* spp.-dominant microbiome may be able to promote HPV clearance or even reverse the process of tumourigenesis, reducing the morbidity resulting from these conditions and their treatments^37^,^38^. Probiotics have been used in a similar manner to reduced recurrence of BV, through accurate, targeted modification of the bacterial community^39^.

Further research is required to understand the molecular mechanisms involved in the complex role that bacterial communities can play in the development of cancer. An understanding of the functional properties of the community state types is required in order to complement what we already know about their structure. Further longitudinal studies are needed to investigate the changes and stability of the microbiome during transition from acute HPV infection, to persistent infection through to development of CIN and cancer.

In summary, this is the first study to correlate the structure of the vaginal microbiome with presence of CIN in women of reproductive age. Our findings suggest that the presence and prevalence of specific vaginal microbiome CSTs may be involved in the pathogenesis of CIN and cervical cancer. We have also identified 5 bacterial species that could help to differentiate low- and high-grade disease, and with further research these may improve our understanding of the role of the bacterial microenvironment in HPV persistence, development of CIN and progression to cancer. Although the development of HPV vaccines will be the main prevention strategy for this disease, its implementation in many settings can be challenging due to financial, cultural barriers and lack of infrastructure. Microbiome modulation with pre- and probiotics towards stable Lactobacillus-dominant vaginal community structure that promotes HPV clearance, could represent low-cost future therapeutic strategies.

Our findings may be of future clinical and therapeutic relevance and raise the clinical question as to whether women with a high diversity vaginal microbiome and cervical pathology should be subject to more intense colposcopic surveillance and/or treatment and whether the examination of the vaginal microbiome could be used as a triage tool for this population. Future longitudinal studies should aim to elucidate the causality between HPV infection, CIN, the immune microenvironment and the vaginal microbiome and increase our understanding of the role that vaginal bacteria in the tumour microenvironment.

## Methods

### Study population – Inclusion and Exclusion criteria

Ethical approval was obtained from the National Research Ethics Service Committee London – Fulham (Approval number 13/LO/0126). All experiments were performed in accordance with the approved guidelines. All patients gave informed consent. We included pre-menopausal non-pregnant women, 18–45 years of age who attended the colposcopy and gynaecology clinics at Imperial College NHS Healthcare Trust. Women were included irrespective of their ethnicity, parity, smoking habits, phase in their cycle and use of contraception. The type of contraception and the time of their cycle (follicular or luteal) were documented. Women who were HIV or hepatitis B/C positive, with autoimmune disorders, who received antibiotics or pessaries within 14 days of sampling, or had a previous history of cervical treatment were excluded. Detailed medical and gynaecological history was collected including time since last sexual intercourse and douching practices. Ethnicity was self-reported as Caucasian, Asian or Black. Histology was used to classify patients into groups. If histology was available from both punch biopsies and treatment cones, the most severe lesion was documented. If histology was not available as not clinically indicated (i.e. healthy controls, low-grade lesions under cytological surveillance), cytology was used for classification.

### Sample collection and processing

A sterile, disposable speculum was inserted, without lubricant, and a swab was taken from the posterior vaginal fornix using a BBL^TM^ CultureSwab^TM^ containing liquid Amies (Becton Dickinson, Oxford, UK) and stored immediately at −80 °C. Whole-Genomic bacterial DNA was extracted from the swabs using a QiAmp Mini DNA kit (Qiagen, Venlo, Netherlands) as previously described [12].

### HPV genotyping

HPV DNA test and 16/18 genotyping was performed according to manufacturer’s guidelines using the Abbott RealTime High Risk (HR) HPV assay on Abbott M2000 platform; a clinically validated *in vitro* polymerase chain reaction (PCR) assay with identification of HPV-16, -18 and 12 other HR HPV subtypes (31, 33, 35, 39, 45, 51, 52, 56, 58, 59, 66, 68)^40^.

### Illumina MiSeq sequencing of 16S rRNA gene amplicons

The V1-V2 hypervariable regions of 16S rRNA genes were amplified by PCR using a forward and reverse fusion primer as previously described^12^. Sequencing was conducted at Research and Testing Laboratory (Lubbock, TX, USA).

### 16S rRNA gene sequence analysis

Sequence data were analysed in Mothur using the MiSeq SOP Pipeline^41^ Sequence reads were quality checked and normalised to the lowest number of reads. Singleton OTUs and OTUs < 10 reads in any sample were collated into OTU_singletons and OTU_rare phylotypes respectively, to maintain normalisation and to minimise artefacts. OTUs were defined using a cut off value of 97% and result data analysed using Vegan package within the R statistical package for assessment of microbial composition and diversity (R Development Core Team 2008). OTU taxonomies (from Phylum to Genus) were determined using the RDP MultiClassifier script to generate the RDP taxonomy^42^ while species level taxonomies of the OTUs were determined using the USEARCH algorithm combined with the cultured representatives from the RDP database^43^. Alpha and beta indices were calculated from these datasets with Mothur and R using the Vegan package.

### Statistical analysis

Subjects were analysed in 4 different phenotype subgroups; normal, LSIL, HSIL and ICC. We included women with HPV changes or ASCUS in the LSIL category and further analysed them separately. Furthermore, we compared HR-HPV positive versus negative women, irrespective of the disease status and also assessed separately women positive for HPV16 versus HPV18 versus other high-risk (negative for HPV16 and HPV18) oncogenic subtypes. Finally, we used data from both the disease and HPV status and compared normal/LSIL HR-HPV negative women, normal/LSIL HR-HPV positive women, HSIL HR-HPV positive and ICC patients.

Analysis of statistical differences between the vaginal microbiome of patient groups was performed using the Statistical Analysis of Metagenomic Profiles (STAMP) package^44^. Data were subjected to multivariate analysis using PCA and HCA by nearest neighbour linkage with a clustering density threshold of 0.75.

To assess potential ascertainment bias of selected clinical characteristics with respect to four phenotype categories of interest, we performed fisher’s exact test for each of the following characteristics (age, ethnicity, parity, smoking, menstrual cycle, contraception and time since last intercourse). To analyse the importance of CSTs with respect to specific phenotype categories, we tested whether CSTs are significantly over or under-represented in any category. For this purpose we created a CST indicator variable, whereby CST = 1 for samples that could be assigned to the given CST and CST = 0 for all other samples. We performed a fisher’s exact test on the corresponding contingency table. Analyses were performed using R and false discovery rate adjustment (Benjamin & Hochberg) was applied to correct p-values. *P*-values and q-values < 0.05 were considered significant.

## Additional Information

**Accession code**: Public access to sequence data and accompanying metadata can be obtained at the European Nucleotide Archive’s (ENA) Sequence Read Archive (SRA) (accession number PRJEB7756).

**How to cite this article**: Mitra, A. *et al*. Cervical intraepithelial neoplasia disease progression is associated with increased vaginal microbiome diversity. *Sci. Rep*. **5**, 16865; doi: 10.1038/srep16865 (2015).

## Supplementary Material

Supplementary Information

## Figures and Tables

**Table 1:** Patient characteristics.

Characteristics	Normal (n = 20)	LSIL (n = 52)	HSIL (n = 92)	Cancer (n = 5)	Total (n = 169)	*P*value	Q value
Age, years							
Mean (SD, range)	31 (5.19, 24–43)	32 (5.66, 23–45)	30 (4.65, 25–43)	34 (5.29, 27–41)	31 (5.08, 23–45)		
Ethnicity, n/N (%)		0.04	0.20				
Caucasian	12/20 (60)	46/52 (88)	78/92 (85)	4/5 (80)	140/169 (83)		
Asian	2/20 (10)	3/52 (6)	9/92 (10)	0/5 (0)	14/169 (8)		
Black	6/20 (30)	3/52 (6)	5/92 (5)	1/5 (20)	15/169 (9)		
Parity, n/N (%)		0.83	1.00				
Nulliparous	16/20 (80)	38/52 (73)	68/92 (74)	3/5 (60)	125/169 (74)		
Parous	4/20 (20)	14/52 (27)	24/92 (26)	2/5 (40)	44/169 (26)		
Smoking status, n/N (%)						0.07	0.37
Current smoker	1/20 (5)	11/52 (21)	28/92 (30)	1/5 (20)	41/169 (24)		
Non-smoker	19/20 (95)	41/52 (79)	64/92 (70)	4/5 (80)	128/169 (76)		
Phase of menstrual cycle, n/N (%)						0.13	0.52
Luteal	7/20 (35)	27/52 (52)	42/92 (46)	5/5 (100)	81/169 (48)		
Follicular	11/20 (55)	21/52 (40)	47/92 (51)	0/5 (0)	79/169 (47)		
Unknown	2/20 (10)	3/52 (8)	3/92 (3)	0/5 (0)	8/169 (5)		
Contraception, n/N (%)						0.02	0.12
Nil	15/20 (75)	18/52 (35)	29/92 (32)	1/5 (20)	63/169 (37)		
Condoms	1/20 (5)	10/52 (19)	16/92 (17)	0/5 (0)	27/169 (16)		
COCP	3/20 (15)	11/52 (21)	35/92 (38)	2/5 (40)	51/169 (30)		
POP	0/20 (0)	4/52 (8)	4/92 (4)	0/5 (0)	8/169 (5)		
Copper IUD	0/20 (0)	2/52 (4)	2/92 (2)	1/5 (20)	5/169 (3)		
Mirena IUS	1/20 (5)	1/52 (2)	3/92 (3)	1/5 (20)	6/169 (4)		
Vaginal Ring	0/20 (0)	0/52 (0)	2/92 (2)	0/5 (0)	2/169 (1)		
Contraceptive implant	0/20 (0)	4/52 (8)	1/92 (1)	0/5 (0)	5/169 (3)		
Contraceptive injection	0/20 (0)	2/52 (4)	0/92 (0)	0/5 (0)	2/169 (1)		
Time since last intercourse, n/N (%)						0.20	0.60
>48 hours	16/20 (80)	49/52 (94)	77/92 (84)	5/5 (100)	147/169 (87)		
<48 hours	4/20 (20)	3/52 (6)	14/92 (16)	0/5 (0)	21/169 (13)		

COCP: Combined oral contraceptive pill; HSIL: High-grade Squamous intraepithelial lesion; IUD: Intrauterine device; IUS: Intrauterine system; LSIL: Low-grade squamous intraepithelial lesion; NR: not relevant; POP: Progesterone-only pill; SD: standard deviation. P-value calculated using Fishers exact test, Q-value calculated using Benjamini-Hochberg false discovery rate (FDR) method.

**Table 2:** Rates of each CST according to disease severity and HPV status/genotype – species level.

	CST I *L. crispatus*n/N (%)	CST II *L. gasseri*n/N (%)	CST III *L. iners*n/N (%)	CST IV *Mixed*n/N (%)	CST V *L. jensenii*n/N (%)	TOTAL n/N (%)
DISEASE SEVERITY (A)						
Normal	10/20 (50)	0/20 (0)	8/20 (40)	2/20 (10)	0/20 (0)	20/20 (100)
LSIL	22/52 (42)	1/52 (2)	12/52 (23)	11/52 (21)	6/52 (12)	52/52 (100)
HSIL	37/92 (40)	3/92 (3)	27/92 (29)	25/92 (27)	0/92 (0)	92/92 (100)
Cancer	1/5 (20)	1/5 (20)	0/5 (0)	2/5 (40)	1/5 (20)	5/55 (100)
*Total*	*70*/*169 (41)*	*5*/*169 (3)*	*47*/*169 (28)*	*40*/*169 (24)*	*7*/*169 (4)*	*169*/*169* (100)
*P* value^1^	0.30	0.12	0.38	0.06	0.47	–
Q value^1^	0.47	0.46	0.47	0.32	0.47	–
DISEASE SEVERITY (B) (ASCUS/LSIL separate)^2^						
Normal	10/20 (50)	0/20 (0)	8/20 (40)	2/20 (10)	0/20 (0)	20/20 (100)
ASCUS	16/26 (62)	0/26 (0)	4/26 (15)	5/26 (19)	1/26 (4)	26/26 (100)
LSIL	6/26 (23)	1/26 (4)	8/26 (31)	6/26 (23)	5/26 (19)	26/26 (100)
HSIL	37/92 (40)	3/92 (3)	27/92 (29)	25/92 (27)	0/92 (0)	92/92 (100)
Cancer	1/5 (20)	1/5 (20)	0/5 (0)	2/5 (40)	1/5 (20)	5/5 (100)
*Total*	*70*/*169 (41)*	*5*/*169 (3)*	*47*/*169 (28)*	*40*/*169 (24)*	*7*/*169 (4)*	*169*/*169* (100)
*P* value^1^	0.10	0.11	0.65	0.06	0.87	–
Q value^1^	0.33	0.33	0.87	0.32	0.87	–
HPV STATUS						
Negative	13/24 (54)	0/24 (0)	6/24 (25)	5/24 (21)	0/24 (0)	24/24 (100)
Positive	38/93 (41)	2/93 (2)	25/93 (27)	26/93 (28)	2/93 (2)	93/93 (100)
*Total*	*51*/*117 (44)*	*2*/*117 (2)*	*31*/*117 (26)*	*31*/*117 (26)*	*2*/*117 (2)*	*117*/*117* (100)
*P* value^3^	0.26	1.00	1.00	0.61	1.00	–
Q value^3^	1.00	1.00	1.00	1.00	1.00	–
HPV GENOTYPE						
HPV-16	13/31 (42)	1/31 (3)	8/31 (26)	9/31 (29)	0/31 (0)	31/31 (100)
HPV-18	3/5 (60)	0/5 (0)	1/5 (20)	1/5 (20)	0/5 (0)	5/5 (100)
Other HR-HPV subtype	12/26 (46)	1/26 (4)	7/26 (27)	5/26 (19)	1/26 (4)	26/26 (100)
*Total*	*28*/*62 (45)*	*2*/*62 (3)*	*16*/*62 (26)*	*15*/*62 (24)*	*1*/*62 (1)*	*62*/*62* (100)
*P* value^3^	0.74	1.00	1.00	0.81	0.50	–
Q value^3^	1.00	1.00	1.00	1.00	1.00	–
DISEASE SEVERITY and HPV STATUS						
Normal/LSIL, HPV neg	11/20 (55)	0/20 (0)	6/20 (30)	3/20 (15)	0/20 (0)	20/20 (100)
Normal/LSIL, HPV pos	17/34 (50)	0/34 (0)	7/34 (21)	7/34 (21)	3/34 (8)	334/4 (100)
HSIL, HPV pos	21/58 (36)	2/58 (3)	16/58 (28)	19/58 (33)	0/58 (0)	58/58 (100)
Cancer	1/5 (20)	1/5 (20)	0/5 (0)	2/5 (40)	1/5 (20)	5/5 (100)
*Total*	*52*/*117 (44)*	*3*/*117 (3)*	*29*/*117 (24)*	*31*/*117 (26)*	*3*/*117 (3)*	*117*/*117* (100)
*P* value^1^	0.08	0.05	0.69	0.15	0.57	–
Q value^1^	0.34	0.24	0.69	0.46	0.69	–

ASCUS: atypical squamous cells of undetermined significance; CST: Community state type; CST I = Lactobacillus crispatus-dominant; CST II = Lactobacillus gasseri-dominant; CST III = Lactobacillus iners-dominant, CST IV = high-diversity, Lactobacillus spp-deplete; CST V = Lactobacillus jensenii-dominant; HPV: Human Papilloma Virus; HPV neg: HPV negative; HPV pos: HPV positive; HR-HPV: High-risk Human Papilloma Virus; HSIL: High-grade Squamous intraepithelial lesion; LSIL: Low-grade squamous intraepithelial lesion.

^1^P-value & Q-value calculated using linear regression.

^2^ASCUS and LSIL are split in two separate groups.

^3^P-value calculated using Fishers exact test, Q-value calculated using Benjamini-Hochberg false discovery rate (FDR) method.
