# Supplementary material for: Cervical intraepithelial neoplasia disease progression is associated with increased vaginal microbiome diversity
Source: Sci Rep. 2015 Nov 17;5:16865. doi: 10.1038/srep16865 (PMC4648063; doi:10.1038/srep16865)
Supplement: Supplementary Information [file srep16865-s1.pdf]

Cervical intraepithelial neoplasia disease progression is associated with increased vaginal microbiome diversity.

Mitra A, MacIntyre DA, Lee YS, Smith A, Marchesi JR, Lehne B, Bhatia R, Lyons D, Paraskevaidis E, Li JV, Holmes E, Nicholson JK, Bennett PR, Kyrgiou M

Supplementary Tables

Supplementary Table 1. Rates of each class-based structure according to disease severity and HPV status/genotype – class level

	**Normal (>97% Bacilli) n/N (%)**	**Transient (85-97% Bacilli) n/N (%)**	**Atypical (<85% Bacilli) n/N (%)**	**TOTAL n/N (%)**
**DISEASE SEVERITY (A)**				
**Normal**	16/20 (80)	2/20 (10)	2/20 (10)	20/20 (100)
**LSIL**	40/52 (77)	4/52 (8)	8/52 (15)	52/52 (100)
**HSIL**	67/92 (73)	4/92 (4)	21/92 (23)	92/92 (100)
**Cancer**	3/5 (60)	0/5 (0)	2/5 (40)	5/5 (100)
Total	126/169 (75)	10/169 (6)	33/169 (19)	*169/169* (100)
***P* value[1]**	0.33	0.21	0.07	-
**Q value[1]**	0.33	0.33	0.20	-
**DISEASE SEVERITY (B) (ASCUS/LSIL separate)[2]**				
**Normal**	16/20 (80)	2/20 (10)	2/20 (10)	20/20 (100)
**ASCUS**	20/26 (77)	2/26 (8)	4/26 (15)	26/26 (100)
**LSIL**	20/26 (77)	2/26 (8)	4/26 (15)	26/26 (100)
**HSIL**	67/92 (73)	4/92 (4)	21/92 (23)	92/92 (100)
**Cancer**	3/5 (60)	0/5 (0)	2/5 (40)	5/5 (100)
Total	126/169 (75)	10/169 (6)	33/169 (19)	*169/169* (100)
***P* value[1]**	0.36	0.23	0.08	-
**Q value[1]**	0.36	0.36	0.25	-
**HPV STATUS**				
**Negative**	17/24 (71)	3/24 (13)	4/24 (17)	24/24 (100)

**Positive**	70/94 (74)	3/94 (3)	21/94 (22)	93/93 (100)
Total	87/118 (74)	6/118 (5)	25/118 (21)	*117/117* (100)
***P* value[3]**	0.80	0.10	0.78	-
**Q value[3]**	1.00	1.00	1.00	-
**HPV GENOTYPE**				
**HPV-16**	22/31 (71)	2/31 (6)	7/31 (23)	31/31 (100)
**HPV-18**	4/5 (80)	0/5 (0)	1/5 (20)	5/5 (100)
**Other HR-HPV subtype**	21/26 (81)	1/26 (4)	4/26 (15)	26/26 (100)
Total	47/62 (76)	3/62 (5)	12/62 (19)	*62/62* (100)
***P* value[3]**	0.81	1.00	0.89	-
**Q value[3]**	1.00	1.00	1.00	-
**DISEASE SEVERITY and HPV STATUS**				
**Normal/LSIL, HPV neg**	16/20 (80)	2/20 (10)	2/20 (10)	20/20 (100)
**Normal/LSIL, HPV pos**	27/34 (79)	1/34 (3)	6/34 (18)	34/34 (100)
**HSIL, HPV pos**	42/58 (72)	1/58 (2)	15/58 (26)	58/58 (100)
**Cancer**	3/5 (60)	0/5 (0)	2/5 (40)	5/5 (100)
Total	88/117 (75)	4/117 (3)	25/117 (22)	*117/117* (100)
***P* value[1]**	0.29	0.10	0.06	-
**Q value[1]**	0.29	0.20	0.19	-

ASCUS: atypical squamous cells of undetermined significance; HPV: Human Papilloma Virus; HPV neg: HPV negative; HPV pos: HPV positive; HR-HPV: High-risk Human Papilloma Virus; HSIL: High-grade Squamous intraepithelial lesion; LSIL: Low-grade squamous intraepithelial lesion

[1]*P-value & Q-value calculated using linear regression.* **[2]**ASCUS and LSIL are split in two separate groups [3]*P-value calculated using Fishers exact test, Q-value calculated using Benjamini-Hochberg false discovery rate (FDR) method.*

Supplementary Table 2. Regression analysis of each class-based structure according to disease severity

Subgroups	Effect	Standard error	t-value	*p*-value	q-value
**DISEASE SEVERITY (A)**					
**Normal**	-0.04	0.05	-0.99	0.33	0.33
**Intermediate**	-0.03	0.02	-1.26	0.21	0.33
**Atypical**	0.08	0.04	1.84	0.07	0.20
**DISEASE SEVERITY (B) (ASCUS/LSIL separate)[1]**					
**Normal**	-0.03	0.03	-0.92	0.36	0.36
**Intermediate**	-0.02	0.02	-1.21	0.23	0.36
**Atypical**	0.05	0.03	1.74	0.08	0.25
**DISEASE SEVERITY and HPV STATUS**					
**Normal**	-0.05	0.05	-1.07	0.29	0.29
**Intermediate**	-0.03	0.02	-1.65	0.10	0.20
**Atypical**	0.09	0.05	1.87	0.06	0.19

[1]ASCUS and LSIL are split in two separate groups

Supplementary Table 3. Regression analysis of each species-based structure according to disease severity

Subgroups	Effect	Standard error	t-value	*p*-value	q-value
**DISEASE SEVERITY (A)**					
**CST I**	-0.05	0.05	-1.05	0.30	0.47
**CST II**	0.03	0.02	1.58	0.12	0.46
**CST III**	-0.04	0.05	-0.88	0.38	0.47
**CST IV**	0.08	0.04	1.87	0.06	0.32
**CST V**	-0.02	0.02	-0.73	0.47	0.47
**DISEASE SEVERITY (B) (ASCUS/LSIL separate)[1]**					
**CST I**	-0.06	0.03	-1.67	0.10	0.33
**CST II**	0.02	0.01	1.60	0.11	0.33
**CST III**	-0.01	0.03	-0.46	0.65	0.87
**CST IV**	0.05	0.03	1.87	0.06	0.32
**CST V**	0.00	0.01	-0.17	0.87	0.87
**DISEASE SEVERITY and HPV STATUS**					
**CST I**	-0.10	0.06	-1.74	0.08	0.34
**CST II**	0.03	0.02	2.01	0.05	0.24
**CST III**	-0.02	0.05	-0.40	0.69	0.69
**CST IV**	0.07	0.05	1.43	0.15	0.46
**CST V**	0.01	0.02	0.57	0.57	0.69

CST: Community state type. [1]ASCUS and LSIL are split in two separate groups

Supplementary Table 4. Richness and Diversity indices for the study population.

	Class Microbiome Status	CST Status	Disease severity (A)	Number of sequences	Species observed	Inverse Simpson	InvSimpson _lci	InvSimpson _hci	Non-parametric Shannon	Coverage
A003	A	4	HSIL	183	12	3.517744	3.097309	4.070248	1.609652	0.983607
A005	N	1	HSIL	183	2	1.068116	1.016053	1.125803	0.144552	1
A006	A	4	HSIL	183	11	4.882146	4.087711	6.059862	1.900957	0.989071
A007	N	5	LSIL	183	1	1	1	1	0	1
A008	N	1	HSIL	183	2	1.01105	0.990009	1.033004	0.055747	0.994536
A009	A	4	HSIL	183	12	5.764278	4.976013	6.849297	1.993397	0.989071
A010	N	3	HSIL	183	4	1.080662	1.023147	1.145029	0.235495	0.989071
A011	N	1	HSIL	183	1	1	1	1	0	1
A013	N	1	LSIL	183	3	1.155896	1.074211	1.251027	0.301669	1
A014	A	4	LSIL	183	22	5.21874	4.167965	6.977929	2.322218	0.961749
A015	N	3	HSIL	183	1	1	1	1	0	1
A017	N	1	LSIL	183	1	1	1	1	0	1
A019	N	3	LSIL	183	1	1	1	1	0	1
A020	A	4	LSIL	183	14	1.585245	1.387612	1.848525	1.102464	0.972678
A021	I	3	NORMAL	183	4	1.602483	1.419498	1.839625	0.727045	0.994536
A022	N	1	NORMAL	183	4	1.548252	1.374855	1.7717	0.694882	1
A024	N	3	NORMAL	183	3	1.104384	1.039173	1.178326	0.234141	0.994536
A026	N	3	LSIL	183	2	1.01105	0.990009	1.033004	0.055747	0.994536
A027	N	4	LSIL	183	3	2.074106	1.997367	2.156977	0.767902	1
A028	N	1	HSIL	183	1	1	1	1	0	1
A029	N	1	HSIL	183	1	1	1	1	0	1

A030	A	4	HSIL	183	11	5.629817	5.030579	6.391123	1.912851	0.989071
A031	N	3	HSIL	183	3	1.092287	1.031066	1.161236	0.217097	0.994536
A032	N	1	HSIL	183	1	1	1	1	0	1
A033	N	3	HSIL	183	3	1.080732	1.023122	1.145217	0.193399	1
A034	I	3	HSIL	183	7	1.398589	1.253024	1.582421	0.704096	0.983607
A035	N	1	HSIL	183	1	1	1	1	0	1
A036	N	3	HSIL	183	1	1	1	1	0	1
A037	N	1	HSIL	183	1	1	1	1	0	1
A038	I	3	HSIL	183	9	1.640043	1.441274	1.902409	0.937286	0.983607
A040	N	1	HSIL	183	2	1.01105	0.990009	1.033004	0.055747	0.994536
A041	N	1	HSIL	183	4	2.073332	1.888526	2.29823	0.863966	0.994536
A042	N	1	HSIL	183	3	1.341361	1.221425	1.487416	0.461393	0.994536
A043	N	3	LSIL	183	3	1.313432	1.198452	1.452815	0.44142	0.994536
A044	N	1	HSIL	183	2	1.022221	0.992476	1.053804	0.067856	1
A045	N	1	HSIL	183	1	1	1	1	0	1
A046	A	4	HSIL	183	13	5.046364	4.268994	6.169878	1.974823	0.989071
A050	A	4	LSIL	183	10	3.794258	3.265046	4.528209	1.626232	0.978142
M001	N	1	HSIL	183	2	1.044927	1.002691	1.090878	0.106675	1
M002	N	3	HSIL	183	5	1.080942	1.023038	1.145796	0.271052	0.983607
M003	N	1	HSIL	183	2	1.352473	1.23242	1.49844	0.427887	1
M004	A	4	HSIL	183	10	3.640796	3.299043	4.061536	1.533157	0.978142
M005	N	3	LSIL	183	3	1.281592	1.166926	1.421248	0.448706	1
M006	N	1	HSIL	183	2	1.01105	0.990009	1.033004	0.055747	0.994536
M009	N	1	LSIL	183	3	1.068459	1.015878	1.126779	0.180856	0.994536
M010	N	3	HSIL	183	2	1.068116	1.016053	1.125803	0.144552	1
M011	A	4	LSIL	183	9	4.074627	3.462428	4.949813	1.679591	0.989071
M012	A	4	HSIL	183	13	7.265707	6.327307	8.530924	2.176772	0.989071
M013	N	3	HSIL	183	2	1.664801	1.517589	1.84364	0.586439	1
M014	N	3	LSIL	183	2	1.033513	0.997027	1.072772	0.08709	1
M015	N	2	LSIL	183	3	1.340066	1.212968	1.496917	0.496449	1

M016	N	1	HSIL	183	1	1	1	1	0	1
M017	N	3	LSIL	183	2	1.380502	1.256105	1.532247	0.446137	1
M018	A	4	LSIL	183	6	3.66	3.195216	4.283019	1.465748	0.994536
M019	N	3	LSIL	183	2	1.165605	1.083625	1.261004	0.270144	1
M020	N	3	HSIL	183	2	1.270155	1.164793	1.396473	0.36736	1
M021	N	1	HSIL	183	2	1.01105	0.990009	1.033004	0.055747	0.994536
M023	A	4	HSIL	183	9	3.574372	2.985317	4.453033	1.626865	1
M024	N	1	HSIL	183	1	1	1	1	0	1
M025	A	4	HSIL	183	10	4.178921	3.556557	5.065301	1.724467	0.994536
M027	N	1	LSIL	183	6	1.336839	1.212055	1.490265	0.580437	0.983607
M028	N	3	HSIL	183	3	1.105116	1.039007	1.18021	0.23109	1
M029	N	1	HSIL	183	2	1.056461	1.009096	1.108493	0.12593	1
M030	N	1	HSIL	183	1	1	1	1	0	1
M031	N	3	HSIL	183	3	1.179474	1.093162	1.280585	0.323981	0.994536
M032	N	1	HSIL	183	5	1.131318	1.05596	1.218258	0.334028	0.994536
M033	N	1	HSIL	183	8	2.852518	2.501651	3.317863	1.314771	0.978142
M034	A	4	HSIL	183	9	2.848127	2.368636	3.571022	1.517152	1
M035	N	1	LSIL	183	4	2.059486	1.881004	2.275389	0.853145	0.994536
M036	N	3	HSIL	183	1	1	1	1	0	1
M038	A	4	HSIL	183	2	1.989368	1.932152	2.050076	0.687748	1
M039	A	4	HSIL	183	7	2.219808	1.939111	2.595525	1.115111	0.989071
M040	N	3	HSIL	183	3	1.068665	1.015776	1.127363	0.174287	1
M041	N	1	HSIL	183	2	1.01105	0.990009	1.033004	0.055747	0.994536
M042	A	4	NORMAL	183	11	1.339312	1.204444	1.508191	0.787372	0.972678
M043	A	4	HSIL	183	1	1	1	1	0	1
M045	N	4	HSIL	183	7	1.647344	1.460551	1.888922	0.835043	0.983607
M046	N	1	LSIL	183	5	2.891648	2.685593	3.13195	1.154027	0.994536
M047	N	3	NORMAL	183	3	1.117801	1.047375	1.198381	0.251092	1
M048	A	4	HSIL	183	3	1.068459	1.015878	1.126779	0.180856	0.994536
M049	N	3	HSIL	183	12	5.050955	4.344906	6.030995	1.906986	0.989071

M050	N	5	LSIL	183	4	1.033706	0.996808	1.07344	0.166376	0.983607
M051	N	1	LSIL	183	2	1.033513	0.997027	1.072772	0.08709	1
M052	N	1	LSIL	183	2	1.338558	1.220785	1.481482	0.41842	1
M054	I	4	HSIL	183	13	2.991915	2.657803	3.422109	1.510216	0.978142
M055	N	5	CANCER	183	12	2.617985	2.193909	3.245289	1.509116	0.989071
M057	N	1	HSIL	183	4	1.105557	1.038906	1.181346	0.260908	1
M058	N	1	NORMAL	183	3	1.033642	0.996881	1.073217	0.123279	0.994536
M059	N	1	HSIL	183	1	1	1	1	0	1
M060	N	3	HSIL	183	2	1.103798	1.039314	1.176813	0.196109	1
M061	N	4	LSIL	183	3	1.033642	0.996881	1.073217	0.123279	0.994536
M062	A	4	HSIL	183	3	1.909967	1.724849	2.139596	0.770555	1
M063	N	1	HSIL	183	13	5.794363	4.939729	7.00659	2.042392	0.983607
M064	A	4	HSIL	183	3	1.068459	1.015878	1.126779	0.180856	0.994536
M065	N	3	HSIL	183	13	5.720715	4.991201	6.699983	1.998711	0.972678
M066	N	1	HSIL	183	2	1.01105	0.990009	1.033004	0.055747	0.994536
M067	N	3	HSIL	183	3	1.578334	1.42611	1.766938	0.597045	1
M068	N	1	LSIL	183	3	1.258635	1.154336	1.383654	0.398258	0.994536
M069	I	4	LSIL	183	1	1	1	1	0	1
M070	N	1	HSIL	183	7	1.256451	1.145423	1.391313	0.551692	0.994536
M071	A	4	HSIL	183	2	1.033513	0.997027	1.072772	0.08709	1
M072	N	3	HSIL	183	8	4.481432	3.903394	5.260426	1.686752	0.994536
M073	A	4	HSIL	183	2	1.01105	0.990009	1.033004	0.055747	0.994536
M074	N	3	LSIL	183	17	8.048816	6.954904	9.551069	2.351875	0.978142
M075	N	3	HSIL	183	3	1.056729	1.008921	1.109295	0.161754	0.994536
M076	N	1	NORMAL	183	1	1	1	1	0	1
M077	N	1	LSIL	183	1	1	1	1	0	1
M078	N	3	HSIL	183	6	1.330537	1.202388	1.489261	0.600545	0.989071
M079	N	1	HSIL	183	2	1.033513	0.997027	1.072772	0.08709	1
M080	N	1	HSIL	183	1	1	1	1	0	1
M081	N	4	HSIL	183	1	1	1	1	0	1

M082	N	1	HSIL	183	3	1.947492	1.836052	2.073333	0.719621	0.994536
M083	N	2	HSIL	183	3	1.25267	1.144884	1.382861	0.420251	1
M084	A	4	HSIL	183	1	1	1	1	0	1
M085	N	5	LSIL	183	5	3.711388	3.443495	4.024481	1.370046	0.994536
M086	N	3	NORMAL	183	2	1.022221	0.992476	1.053804	0.067856	1
M087	A	4	LSIL	183	3	1.205429	1.11284	1.314823	0.350183	0.994536
M088	I	5	LSIL	183	4	1.461815	1.312673	1.649192	0.609267	0.994536
M089	A	4	LSIL	183	4	1.419451	1.283331	1.587873	0.555176	0.994536
M090	N	1	LSIL	183	6	1.268897	1.156058	1.406146	0.528551	0.994536
M091	N	2	HSIL	183	2	1.022221	0.992476	1.053804	0.067856	1
M093	N	3	HSIL	183	2	1.01105	0.990009	1.033004	0.055747	0.994536
M094	N	1	HSIL	183	1	1	1	1	0	1
M095	A	4	HSIL	183	2	1.022221	0.992476	1.053804	0.067856	1
M096	N	1	LSIL	183	10	1.480925	1.310713	1.701943	0.886108	0.989071
M097	N	1	HSIL	183	2	1.068116	1.016053	1.125803	0.144552	1
M100	N	1	HSIL	183	1	1	1	1	0	1
M101	I	3	LSIL	183	2	1.01105	0.990009	1.033004	0.055747	0.994536
M102	N	3	LSIL	183	2	1.217058	1.122814	1.328571	0.321466	1
M103	N	2	HSIL	183	1	1	1	1	0	1
M104	N	5	LSIL	183	1	1	1	1	0	1
M132	N	1	CANCER	183	3	2.192049	1.93465	2.528453	0.923912	1
M133	N	3	LSIL	183	1	1	1	1	0	1
M136	N	5	LSIL	183	2	1.297266	1.186741	1.430492	0.388562	1
M138	N	1	NORMAL	183	4	1.06887	1.015673	1.127949	0.209686	0.994536
M140	N	1	NORMAL	183	2	1.165605	1.083625	1.261004	0.270144	1
M141	N	3	NORMAL	183	3	1.033642	0.996881	1.073217	0.123279	0.994536
M142	N	1	NORMAL	183	3	1.49946	1.357499	1.67458	0.555182	0.994536
M143	N	3	NORMAL	183	4	1.056931	1.008791	1.109895	0.197294	0.989071
M145	N	3	NORMAL	183	1	1	1	1	0	1
M152	N	1	LSIL	183	2	1.068116	1.016053	1.125803	0.144552	1

M153	N	1	NORMAL	183	2	1.01105	0.990009	1.033004	0.055747	0.994536
M154	A	4	NORMAL	183	7	2.209207	1.908653	2.622107	1.127411	1
M156	N	1	LSIL	183	2	1.678561	1.531484	1.856887	0.59171	1
M157	N	1	HSIL	183	6	1.703457	1.524084	1.930686	0.787029	0.989071
M158	N	1	NORMAL	183	2	1.091785	1.031222	1.159906	0.179617	1
M159	N	1	LSIL	183	3	2.020505	1.876927	2.187869	0.775368	1
M160	N	1	LSIL	183	1	1	1	1	0	1
M162	N	1	LSIL	183	2	1.091785	1.031222	1.159906	0.179617	1
M163	N	1	LSIL	183	2	1.01105	0.990009	1.033004	0.055747	0.994536
M164	N	1	LSIL	183	4	2.138839	1.947998	2.371133	0.893032	0.994536
M166	N	3	NORMAL	183	4	1.35832	1.233834	1.510745	0.513787	0.989071
M168	N	3	LSIL	183	3	1.261878	1.154863	1.390751	0.406843	1
M169	N	1	LSIL	183	2	1.01105	0.990009	1.033004	0.055747	0.994536
M170	A	4	LSIL	183	10	1.214661	1.113675	1.335787	0.618425	0.967213
M174	A	4	CANCER	183	7	4.976987	4.590058	5.435157	1.676453	0.994536
M175	N	3	HSIL	183	6	1.158228	1.074144	1.256594	0.400836	0.994536
M176	A	4	CANCER	183	16	6.218447	5.25611	7.612147	2.175393	0.983607
M177	A	4	HSIL	183	16	7.056356	6.129191	8.314021	2.230323	0.972678
M178	I	1	NORMAL	183	7	2.935484	2.548075	3.461818	1.331454	0.989071
M180	N	1	LSIL	183	3	1.033642	0.996881	1.073217	0.123279	0.994536
M181	N	2	CANCER	183	1	1	1	1	0	1
M182	N	1	HSIL	183	2	1.566162	1.421882	1.74303	0.545215	1
M183	N	3	HSIL	183	2	1.091785	1.031222	1.159906	0.179617	1
M185	N	1	NORMAL	183	3	1.068459	1.015878	1.126779	0.180856	0.994536
M186	N	3	HSIL	183	2	1.044927	1.002691	1.090878	0.106675	1
M187	N	4	HSIL	183	3	1.818609	1.66626	2.00162	0.688203	1
M188	N	1	LSIL	183	1	1	1	1	0	1
M189	N	3	HSIL	183	1	1	1	1	0	1

A: Abnormal; CST: Community state type; CST I = Lactobacillus crispatus-dominant; CST II = Lactobacillus gasseri-dominant; CST III = Lactobacillus iners-dominant, CST IV = high-diversity, Lactobacillus spp-deplete; CST V = Lactobacillus jensenii-dominant; HSIL: High-grade squamous intraepithelial lesion; I:

Intermediate; InvSimpson_hci: Inverse Simpson higher confidence interval; InvSimpson_lci: Inverse Simpson lower confidence interval; LSIL: Low-grade squamous intraepithelial lesion; N: Normal

Supplementary Figures

Supplementary Figure 1. Bacterial class diversity in study cohort and controls

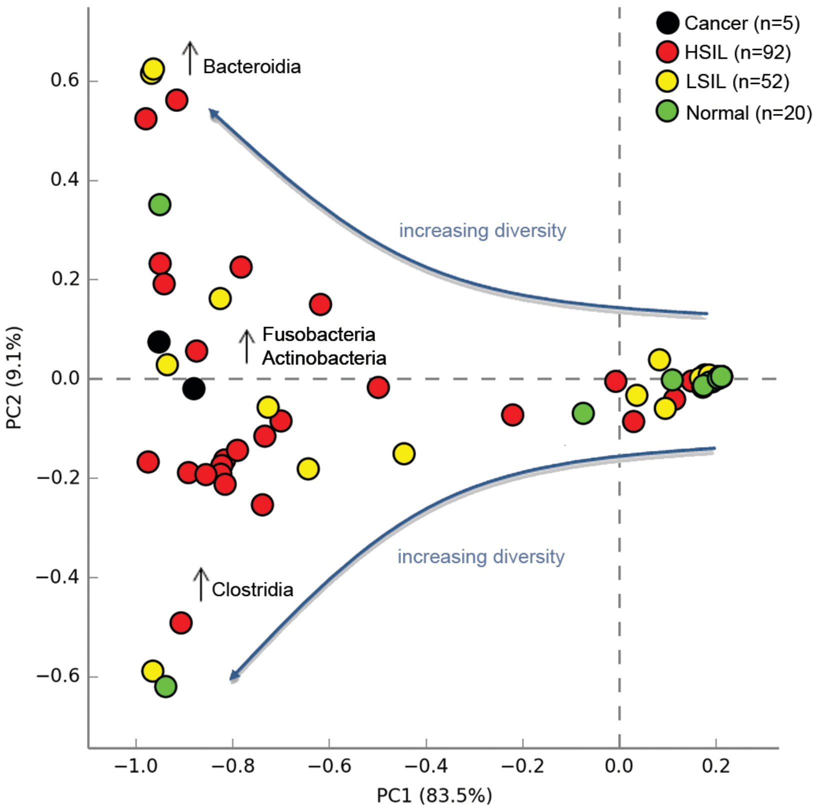

KEY - Cancer: Black; High-grade squamous intra-epithelial lesions (HSIL): Red; Low-grade squamous intra-epithelial lesions (LSIL): Yellow; Normal: Green.

Supplementary Figure 2. Vaginal microbiome composition according to disease and HPV status Hierarchical clustering analysis (HCA) using centroid clustering.

DISEASE SEVERITY I - Normal: Green; Low-grade squamous intra-epithelial lesions (LSIL): Yellow; High-grade squamous intra-epithelial lesions (HSIL): Red; Cancer: Black

DISEASE SEVERITY II - Normal: white; ASCUS: light grey; LSIL: dark grey; HSIL: blue; Cancer: pink

HPV STATUS - HPV negative: light grey; HPV positive: blue; Unknown: white

HPV GENOTYPE - HPV negative: light grey; HPV16: pink; HPV18: blue; Other HR-HPV: dark grey; Unknown: white

DISEASE SEVERITY I & HPV STATUS - LSIL/Normal HPV negative: light grey; LSIL/Normal HPV positive: dark grey; HSIL/HPV positive: blue; Cancer: pink; unknown/other: white

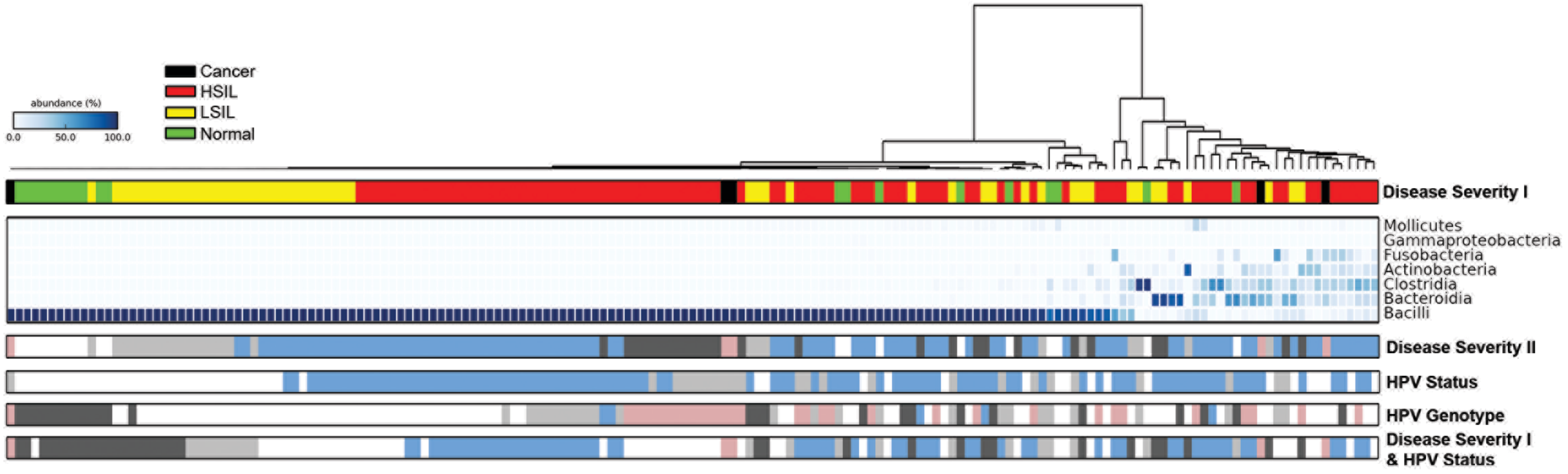

KEY - ASCUS: atypical squamous cells of undetermined significance; CST: community state type; HPV: Human Papillomavirus; HR-HPV: high-risk HPV; HSIL: High-grade squamous intra-epithelial lesion; ICC: invasive cervical cancer; LSIL: Low-grade squamous intra-epithelial lesion

Supplementary Figure 3. Mean proportion of *Lactobacillus* spp. (A) content and *Snethia* spp. (B) content according to disease severity (normal vs LSIL vs HSIL vs ICC)

A.

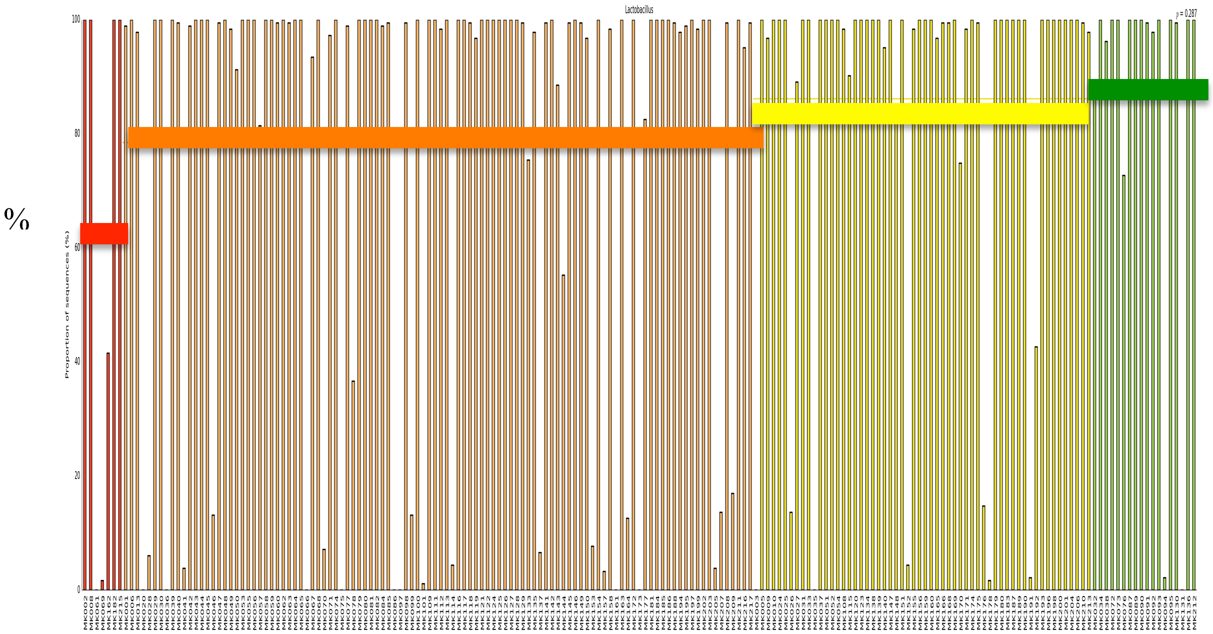

B.

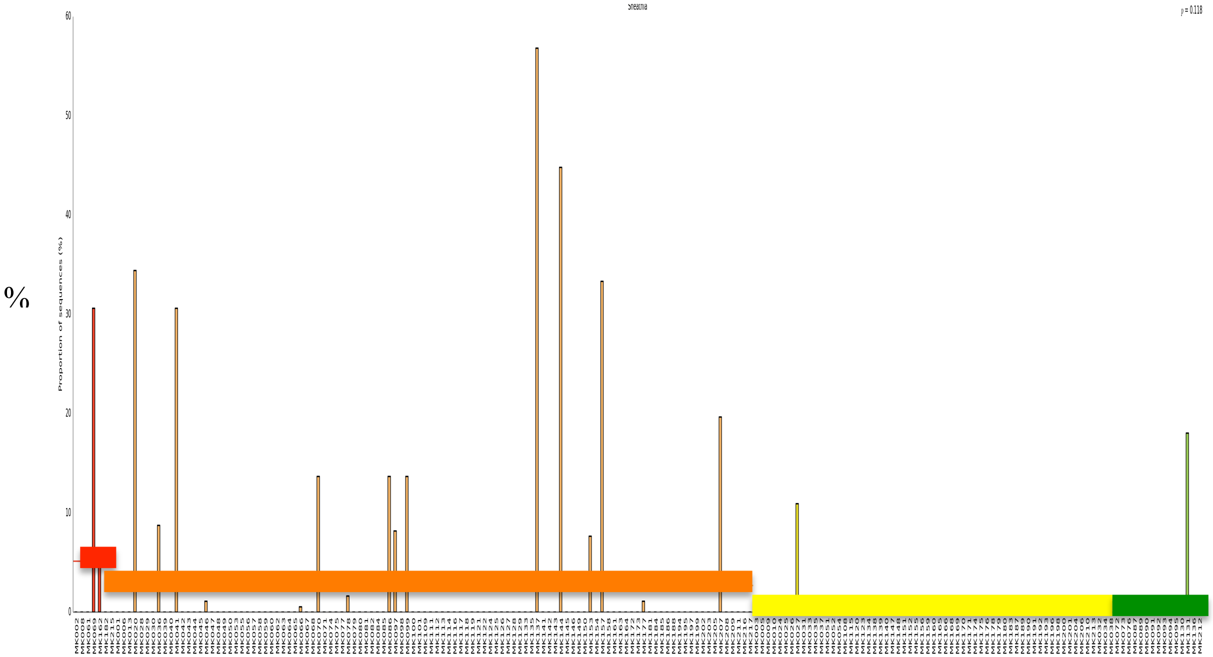

KEY: Normal: Green; Low-grade squamous intra-epithelial lesions (LSIL): Yellow; High-grade squamous intra-epithelial lesions (HSIL): Orange; Invasive Cervical Cancer (ICC): Red
